# Hypersensitivity to triforine in lettuce is triggered by a TNL gene through the disease‐resistance pathway

**DOI:** 10.1111/pbi.13679

**Published:** 2021-08-25

**Authors:** Guanghui An, Ivan Simko, Jiongjiong Chen, Changchun Yu, Dean Lavelle, Weiyi Zhang, Richard W. Michelmore, Hanhui Kuang

**Affiliations:** ^1^ Key Laboratory of Horticultural Plant Biology Huazhong Agricultural University Wuhan China; ^2^ U.S. Department of Agriculture Agricultural Research Service U.S. Agricultural Research Station, Crop Improvement and Protection Research Unit Salinas CA USA; ^3^ Genome Center and Department of Plant Sciences University of California Davis CA USA

**Keywords:** triforine, lettuce, hypersensitive response, *R*‐gene, ROS burst

The majority of cloned disease‐resistance genes (*R*‐genes) encode proteins with nucleotide‐binding leucine‐rich repeat domains (NLRs). *R*‐genes tend to be physically clustered, and the structure of the cluster often facilitates expansion and sequence exchange amongst *R‐*gene homologues (Luo *et al*., 2012). NLR proteins interact directly or indirectly with pathogens effectors, often triggering programmed cell death, also known as hypersensitive response (HR) at the infected sites (Dangl and Jones, 2001). HR may be triggered by pesticide molecules rather than pathogen effectors. For example, some tomato cultivars are sensitive to fenthion, developing toxic lesions after exposure to fenthion (Martin *et al*., 1994). Similarly, some lettuce germplasms are highly sensitive to triforine, an active ingredient in some commercial fungicides, with leaves showing wilting and necrosis 24 h after exposure to triforine (Figure 1a). Sensitivity to triforine in lettuce is controlled by a single locus (*Tr*) (Simko *et al*., 2011); however, the causal gene and its molecular mechanism remain unknown.

**Figure 1:**
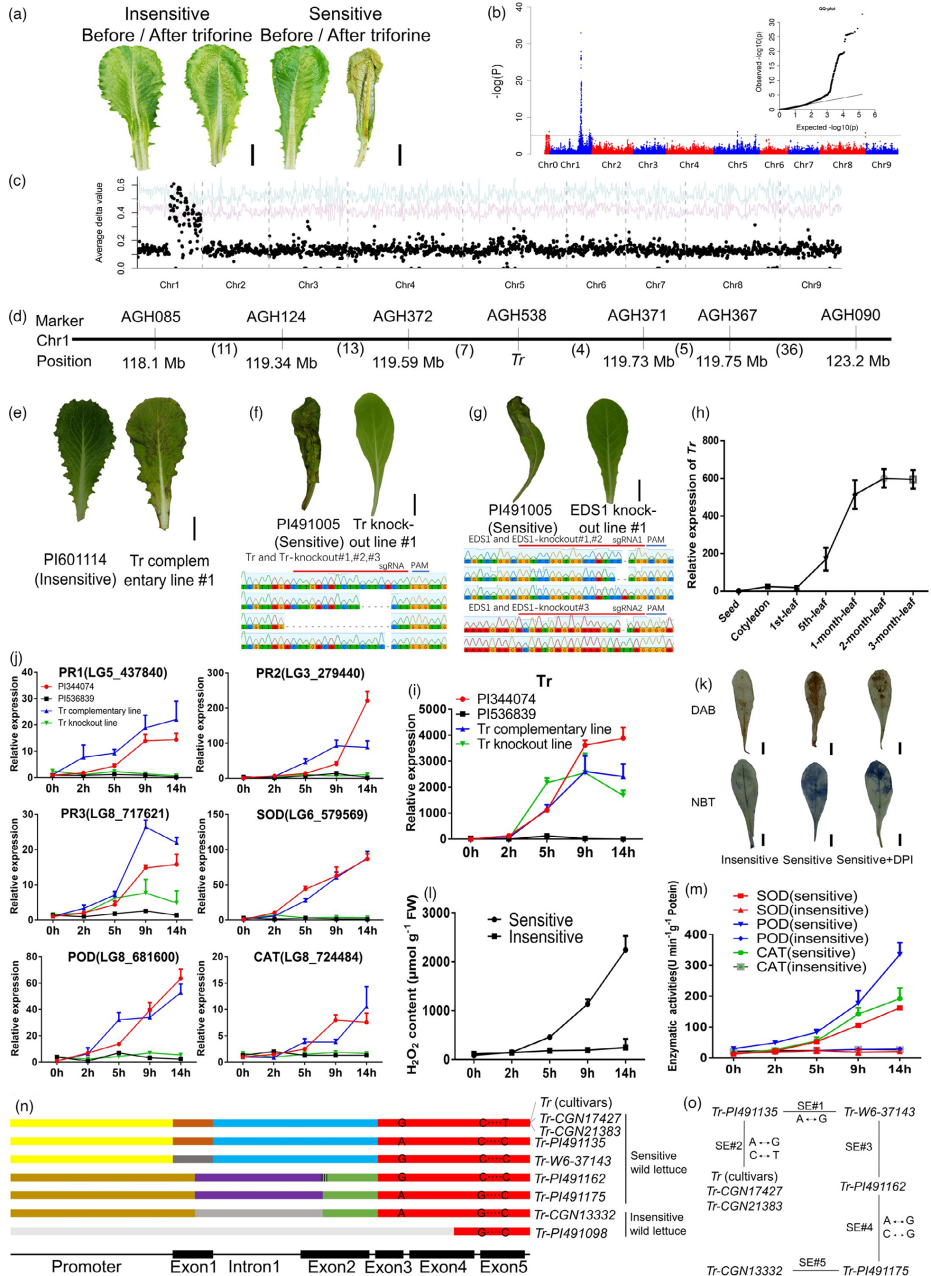
*Tr* triggered hypersensitivity to triforine in lettuce. (a) Detached leaves from the one‐month‐old, sensitive and insensitive cultivars before/after triforine treatment. (b) Genome‐wide association study of the sensitivity to triforine in lettuce. An R script was used to generate quantile‐quantile plots. A significant signal is shown on linkage group 1. (c) BSR‐seq analysis of the *Tr* gene. The Δvalue was plotted along with the nine linkage groups of lettuce (*X*‐axis). The red and green curves refer to confidence intervals of *P* = 0.05 and *P* = 0.01, respectively. (d) The *Tr* gene was fine mapped to 140 kb region. The numbers below the horizontal line refer to the number of recombinants between two markers from 4639 progenies. (e) The complementation test changed plants insensitive to triforine to sensitive. All *Tr* gene (f) and *EDS1* gene (g) knock‐out lines caused frame‐shift deletions, resulting in the phenotypic change from sensitive to insensitive. (h–j) The expression level of the *Tr* gene and other genes associated with disease resistance. (k–m) Sensitive genotypes have stronger ROS than insensitive genotypes when treated with triforine. ‘Sensitive + DPI’ refers to sensitive genotypes pretreated with diphenyleneiodonium chloride. Mean and standard deviation values were calculated using three biological replicates. (n–o) Reconstruction of sequence exchange events amongst *Tr* and its six homologues. Rectangle with the same colour represents identical sequences.

In this study, we used genome‐wide SNPs to perform genome‐wide association studies (GWAS) on sensitivity to triforine. The results showed a significant signal on chromosome 1 (Chr1) (Figure 1b). The candidate region spans 5087 kb and the prominent candidate genes include a TNL‐encoding family. To confirm the GWAS results and to clone the *Tr* gene, we constructed an F_2_ population by crossing a sensitive genotype (PI344074, Romaine) with an insensitive genotype (PI536839, Crisphead). Using Bulk Segregant Analysis + RNA‐seq, the *Tr* gene was mapped to Chr1 (Figure 1c). Then, a total of 4639 individuals from an F_3_ family were first screened using two far‐end flanking markers and recombinants were further genotyped using markers in the candidate region. The *Tr* gene was ultimately fine mapped between markers AGH372 (F‐primer:AACTTGACATTCTTCGGTG/R‐primer:CTTCTGTTTAGTACAACATT) and AGH371 (F‐primer:TTTAGATACCTATGACAACTT/R‐primer:GTATATGTATCTATGTCTATGT), with an interval of approximately 140 kb (Figure 1d). This region of the reference genome (*Lactuca sativa* cv Salinas V8) has five genes, and all of them belong to a TIR type NLR‐encoding family (TNL). Thus, we hypothesize that the *Tr* gene in sensitive parents was a homologue of this *R*‐gene family.

To obtain the *Tr* gene, we used conserved primers to PCR amplify homologues of the *R*‐gene family from the two parental genotypes, PCR products were cloned, and individual colonies were sequenced. Twenty‐one and nine distinct *Tr* homologues were obtained from the sensitive and insensitive parents, respectively. Markers specific to each homologue were designed and used to screen the recombinants. The genetic analysis showed that only one (*Tr‐like109*) homologue from the sensitive parent co‐segregated with sensitivity to triforine. We also used the same pair of conserved primers to amplify homologues of the *R*‐gene family from 29 sensitive accessions, PCR products were pooled and sequenced using Illumina Hiseq2500 platform. Similarly, PCR products from 46 insensitive individuals were also sequenced. Nine SNPs specific to the sensitive pool were found, which all present in the homologue *Tr‐like109*. Therefore, the *Tr‐like109* gene is very likely the candidate for the *Tr*. Indeed, transformation of the *Tr‐like109* gene into the insensitive accession changed its reaction to triforine from insensitive to sensitive in the transformants (Figure 1e). On the other hand, three CRISPR knock‐out lines of the *Tr‐like109* gene in the sensitive accession changed its sensitive reaction to insensitive, confirming that *Tr‐like109* is the *Tr* gene encoding sensitivity (susceptibility) to triforine (Figure 1f).

*Tr* transcripts were detected at multiple developmental stages in sensitive individuals. The expression of the *Tr* gene increased with leaf age in the first month after germination, then the increase slowed and the expression peaked in the second month, and maintained a high expression level for at least one more month (Figure 1h). We also analysed the expression of genes associated with disease resistance in parents, complementary and knock‐out line after treatment with triforine. The *Tr* gene was rapidly up‐regulated after treatment with triforine, and similar upregulation was also found for some genes associated with disease‐resistance response (Figure 1i–j).

To verify that the sensitive response to triforine followed the same pathway as the HR in disease‐resistance, we knocked out the *EDS1* (*LG1_140621*) gene in the sensitive accession, which is required in the resistance pathway for TNL proteins. The homozygous *eds1* mutants were insensitive to triforine (Figure 1g). We, therefore, conclude that the *Tr* gene triggers the HR response through the disease‐resistance pathway. Next, we investigated whether the accumulation of ROS was associated with sensitivity to triforine. DAB, NBT as well as H_2_O_2_ content showed that the accumulation of ROS in the leaves originating from triforine‐sensitive individuals were higher than leaves originating from triforine‐insensitive individuals after triforine treatment (Figure 1k–l). The enzymatic activities of superoxide dismutase (SOD), peroxidase (POD) and catalase (CAT) were considerably higher in the sensitive genotype than in the insensitive genotypes (Figure 1m). Sensitivity to triforine was alleviated if the sensitive plants were pretreated with the ROS‐inhibitor diphenyleneiodonium chloride (Figure 1k).

We used primers specific to the *Tr* gene to amplify PCR products from 817 lettuce accessions (203 cultivated lettuce (*Lactuca sativa*) and 614 wild lettuce (*Lactuca serriola*)). As expected, PCR products were obtained from all sensitive accessions, including 26 cultivated and six wild accessions. PCR products were also obtained from two insensitive accessions. The *Tr* sequences from all sensitive lettuce cultivars and two sensitive *L. serriola* accessions (CGN17427, CGN21383) were identical (Figure 1n). Therefore, the *Tr* gene likely originated from *L. serriola* and underwent at least five sequence exchanges (SE) during domestication or introgression (Figure 1o).

In this study, the *Tr* gene was identified through GWAS and map‐based cloning. The candidate region contains a large *R*‐gene family, which made the identification of the candidate gene challenging. We exhaustively sequenced the *R*‐gene family in the two parents, and a large segregating population was used to narrow down the candidate gene, which facilitated the following process of gene verification. The *Tr* gene, encoding extreme sensitivity to triforine, has potential applications in plant biotechnology. For example, the *Tr* gene, if included in a transformation vector, can be used for larger‐scale selection for marker‐free individuals. It can be also used as a selection marker in mutagenesis to study the HR signalling transduction pathway.

## Accession numbers

The data sets are available in the NCBI (PRJNA689977). The sequences of the *Tr* gene and its homologues are available in GenBank (MW451218‐MW451224).

## Conflict of interest

The authors declare no conflict of interest.

## Author contributions

H.K. designed the project. G.A. performed the experiments. I.S., J.C. and C.Y. performed initial tests. W.Z. provided bioinformatics support. D.L. and R.W.M provided genomics support. G.A. wrote the manuscript with the help of H.K., I.S. and R.W.M.
